# On the non-uniqueness problem in integrated information theory

**DOI:** 10.1093/nc/niad014

**Published:** 2023-06-24

**Authors:** Jake R Hanson, Sara I Walker

**Affiliations:** School of Earth and Space Exploration, Arizona State University, Tempe, AZ, USA; Beyond Center for Fundamental Concepts in Science, Arizona State University, Tempe, AZ, USA; Association for Mathematical Consciousness Science, Munich Center for Mathematical Philosophy, Munich, BY, Germany; School of Earth and Space Exploration, Arizona State University, Tempe, AZ, USA; Beyond Center for Fundamental Concepts in Science, Arizona State University, Tempe, AZ, USA; ASU-SFI Center for Biosocial Complex systems, Arizona State University, Tempe, AZ, USA; Santa Fe Institute, Santa Fe, NM, USA; Association for Mathematical Consciousness Science, Munich Center for Mathematical Philosophy, Munich, BY, Germany

**Keywords:** integrated information theory (IIT), consciousness, non-uniqueness, computational neuroscience, mathematical theories of consciousness

## Abstract

Integrated Information Theory (IIT) 3.0 is among the leading theories of consciousness in contemporary neuroscience. The core of the theory relies on the calculation of a scalar mathematical measure of consciousness, Φ, which is inspired by the phenomenological axioms of the theory. Here, we show that despite its widespread application, Φ is not a well-defined mathematical concept in the sense that the value it specifies is non-unique. To demonstrate this, we introduce an algorithm that calculates all possible Φ values for a given system in strict accordance with the mathematical definition from the theory. We show that, to date, all published Φ values under consideration are selected arbitrarily from a multitude of equally valid alternatives. Crucially, both $\Phi=0$ and $\Phi\gt0$ are often predicted simultaneously, rendering any interpretation of these systems as conscious or not, non-decidable in the current formulation of IIT.

## Introduction

Integrated Information Theory (IIT) is a leading contender among contemporary theories of consciousness^1^. What sets it apart from other contemporary theories such as Global Neuronal Workspace Theory Dehaene and Changeux (2004) or Predictive Processing Dolkega and Dewhurst (2020); Wilkinson et al. (2019); Seth (2014); Hobson et al. (2014); Hohwy (2018) is IIT’s commitment to mathematical rigor and the potential to make quantitative predictions because of this. Epistemologically, the theory is grounded in a phenomenology-first approach Negro (2020), meaning that the theory starts with phenomenological axioms and, from these, deduces a mathematical measure.

The process of deducing a mathematical measure from the axioms of the theory has two key components: first, the phenomenological axioms must be translated into physical postulates and second, the physical postulates must be translated into well-defined mathematical objects. In both steps, there is a chance for the definition of Φ to lose specificity. The first arises because the map from phenomenological axioms to physical postulates may not be unique, meaning that the phenomenological axioms of the theory must be revised in order to provide necessary and sufficient conditions that lead to a precise physical formulation. Similarly, it is possible that many different mathematical objects capture the physical processes that embody consciousness. In both cases, the problem is that the phenomenological axioms of the theory are not precise enough to constrain a detailed physical or mathematical description (or at minimum a class of mathematical forms that are shown to yield equivalent predictions). Conversely, the level of specificity required for mathematical rigor has not yet found rigid justification in terms of phenomenology: mathematical differences such as the choice of distance measure (Wasserstein distance or Kullback–Leibler divergence) do not easily translate into descriptions of the subjective differences they intend to capture.

Critiques of IIT focus on different aspects of this problem. For example, Barrett et al. point out that the mathematical definition of Φ is insufficiently constrained by the postulates of the theory Barrett and Mediano (2019), while McQueen McQueen (2019) highlights an invalid inference procedure from axioms to postulates. In addition, there are many critiques of the axioms themselves Cerullo (2015); Mindt (2017); Bayne (2018), which demonstrate the difficulty in trying to specify phenomenological axioms for consciousness, even without the rigor required by a mathematical formalism. Here, we present a fundamentally different type of ambiguity. Rather than focusing on the translation from phenomenology to postulates or postulates to mathematics, we accept the axioms, postulates, and mathematical definition of the theory at face value and show that the Φ values that result are neither unique nor specific. The values are non-unique in the sense that a multitude of values result from the precise application of the theory, and the values are non-specific in the sense that the possible values are not confined to a small portion of the range. We demonstrate the extent of this problem by calculating all possible Φ values for a corpus of small systems, with the aim of providing context to existing results and emphasizing the importance of avoiding non-unique algorithms for IIT moving forward, particularly in efforts to test its predictions across various experimental systems.

## The non-uniqueness problem

The observation that Φ is indeterminate for some systems has been known since as early as 2012 Tononi (2012). This indeterminacy arises as a consequence of what has occasionally been referred to as “underdetermined qualia” or “tied purviews” in the IIT literature Krohn and Ostwald (2017); Moon (2019). Yet, the extent to which this problem must be addressed in order to interpret already published Φ values remains largely unexamined. In Section “Mathematical Details”, we provide a detailed mathematical description of the issues surrounding non-uniqueness. To summarize here, the challenge is that Φ (“big phi”) is the result of a minimization routine that is not guaranteed to produce a unique value. One applies a scalar function *ϕ* (“little phi”) to an ensemble of probability distributions (called “causes” and “effects”) and selects the distribution with the smallest *ϕ* value. However, if multiple distributions in the ensemble have the same *ϕ* value, which is often the case, there is no prescription to decide which distribution to carry forward in the calculation. Since the final result is sensitive to this choice of distribution, a multitude of Φ values can result from the calculation.

Despite its importance, the problem of degenerate causes/effects remains under-addressed. Moon (2019) is a notable exception. To our knowledge, there are only four proposed solutions to this problem Oizumi et al. (2014); Krohn and Ostwald (2017); Moon (2019) and they differ drastically in content.

The first solution was put forth by Oizumi et al. (2014), where in Figure S1 of their Supporting Information, the authors suggest that the degenerate cause corresponding to the biggest purview element should be selected as the unique cause. Note that a purview element is simply a subset of the system for which a cause/effect is calculated (Section “Mathematical Details”). The justification for this argument rests on the assumption that a larger purview “specifies information about more system elements for the same value of irreducibility” Oizumi et al. (2014); in other words, the *ϕ* values of the degenerate causes/effects may be the same, but bigger purview elements constrain more of the system and should therefore be selected. However, as pointed out by Moon Moon (2019), this justification does not derive from the postulates of IIT and alternative solutions are equally consistent with the phenomenology. Furthermore, there is no guarantee that the largest purview element is unique, as “tied purview elements are often the same size”. Thus, this cannot be a general resolution to the problem. Instead, it is a specific solution that yields a unique Φ value for the system under consideration by Oizumi et al. (2014). It is worth noting that this approach is adopted by Maynard et al. in the implementation of the Python package PyPhi  Mayner et al. (2018), which is widely used to calculate Φ in practice. That said, the computational implementation of the algorithm also fails to address what to do in the case of tied purview elements of the same size and defaults to selecting the first of the elements under consideration.

The second proposed solution, introduced by Krohn and Ostwald (2017) (hereafter KO17), is the opposite of that proposed by Oizumi et al. Instead of selecting the largest purview element, KO17 argues that it is the smallest purview element that should be selected as the core cause/effect in the case of tied purviews. Their motivation stems from a central idea in IIT that “causes should not be multiplied beyond necessity” Tononi (2012). This point is crucial in justifying IIT’s exclusion postulate but was not originally intended for application to the dimensionality of tied purview elements. More importantly, this proposed solution also does not address if the tied purview elements are the same size, which means that it does not guarantee a mathematically well-defined value of Φ.

To remedy this, KO17 presents a third solution based on a modified version of Φ that takes the sum of the degenerate values rather than arbitrarily selecting, or imposing a rule to select, just one. The benefit of this modified definition is that it always results in a single Φ value, but the downside is that it does not account for the shape of the probability distribution attached to the selected cause/effect, which is central to the standard interpretation of IIT. Without carrying the probability distribution forward in the calculation, there is no longer any notion of concepts in qualia space or quales in general Haun and Tononi (2019).

**Figure 1.**
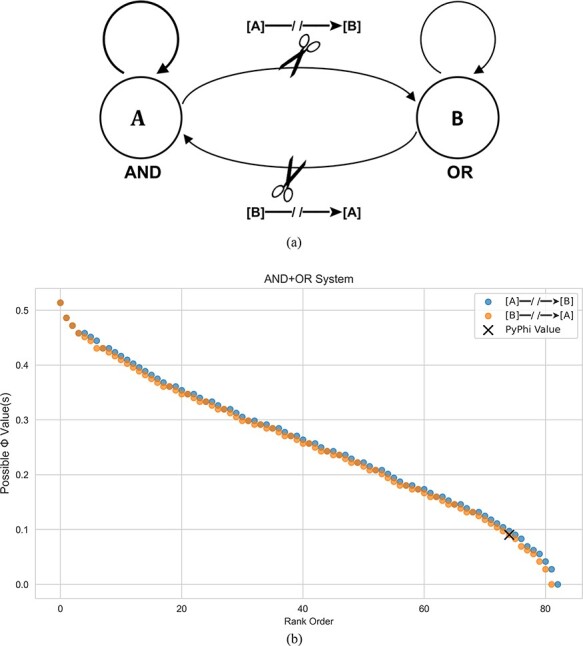
(a) A simple system comprising fully connected AND+OR logic gates. Nodes are labeled as *A* and *B*. Partitions are calculated by cutting the connection from one element to the other in a unidirectional fashion. (b) The spectrum of Φ values that result from each cut of the AND+OR system. The single Φ value output from the Python package PyPhi is shown as an “x”.

The last proposed solution is the “differences that make a difference” criterion put forth by Moon Moon (2019). Here, the author argues that if degenerate causes/effects exist, then none of the corresponding purview elements should be selected. This solution is based on the idea that in IIT to exist is to cause a difference “from the intrinsic perspective of the system”, which is quantified by *ϕ*. If tied purview elements exist with the same *ϕ* value, then it is possible to eliminate one of the elements without changing the *ϕ* value and therefore that element does not exist from the intrinsic perspective of the system. For example, if the purview elements *A*^*p*^ and *B*^*p*^ both give rise to $\phi=1/6$, one can eliminate *B*^*p*^ without changing the *ϕ* value from $1/6$; therefore, the existence of *B*^*p*^ does not make a meaningful difference according to *ϕ*. If one repeats this argument in turn for each of the degenerate causes/effects, they are forced to conclude that each fails to exist from the intrinsic perspective of the system and, consequently, none of the tied purview elements can be selected. While this approach is perhaps the most principled, it leads to $\Phi=0$ for systems that are clearly integrated. For example, if we apply the Moon definition of Φ to a fully connected AND+OR logic gate system, such as that shown in Figure 1(a), we find $\Phi=0$ despite the fact that both logic gates are integrating information from disparate inputs. Put simply, cutting the input to an AND gate does make a difference in terms of its ability to integrate information, and a measure of integrated information that does not account for this should be cause for questioning.

To summarize, challenges in the calculations of Φ and its uniqueness arise with all four existing solutions to the non-uniqueness problem. In the case of selecting the smallest or largest purview element, we are not guaranteed a unique mathematical solution due to the fact that tied purviews are often the same size. In addition, the justification as to why we should select either the smallest or largest element poses challenges when connecting back to the core phenomenology of IIT. Conversely, the modified definitions of Φ put forward by Moon and KO17 do produce unique values but violate expectations of what Φ is designed to measure. In the case of the KO17 definition of Φ, we throw away crucial information about the shapes of the underlying distributions, which underpin the notion of qualia in IIT. Similarly, the Moon definition is overly restrictive in that it often yields $\Phi=0$ for systems that are clearly integrating information, such as a fully connected AND gate.

## Methodology

In this section, we identify and address the source of non-uniqueness in IIT 3.0. We assume that the reader is familiar with the basics of the theory, as presented in Oizumi et al. (2014), although for our purposes it will suffice to understand that the final Φ value is the result of a nested sequence of six different min/max operations, as shown in Algorithm 1. Each min/max optimization yields a different measure of integrated information, which means that there are six different measures to keep track of *ϕ*, $\phi^{Max}_{cause/effect}$, *ϕ*^*Max*^, Φ, $\Phi^{MIP}$, and $\Phi^{Max}$. Note that it is the outermost measure—$\Phi^{Max}$ —that corresponds to the overall level of consciousness according to theory; however, this last optimization step is often neglected with the understanding that one is typically only interested in a specific subsystem, rather than the system as a whole. This is because considering the system as a whole is both computationally intractable (see Section “On the computational complexity of Φ”) and philosophically challenging in all but the simplest cases Hoel et al. (2016). For these reasons, it is the $\Phi^{MIP}$ value, rather than the $\Phi^{Max}$ value, that is typically published, as this is the end result of most calculations and the standard output from PyPhi. In practice, the superscript is usually dropped as a matter of convenience, leaving the reader to infer from context whether “Φ” refers to $\Phi^{MIP}$, $\Phi^{Max}$, or just Φ. Fortunately, this difference between Φ values rarely makes a difference in terms of understanding, since the three measures are closely related conceptually. In our results, the degeneracy occurs well before both the Φ and $\Phi^{MIP}$ calculations, so both suffer from the same problem.

**Algorithm 1:** pseudocode overview of the routine to calculate $\Phi^{Max}$

*## Calculate Phi_Max*

for each subsystem in the powerset of system elements:

 *## Calculate Phi_MIP*

 for each unidirectional partition of the subsystem:

  *## Build the Cause-Effect Structure (CES)*

  for each mechanism in the powerset of the subsystem elements:

   *## Calculate phi_max*

   for each element in the past purview:

    *## Calculate phi_cause_max*

    for every partition of the purview element:

     *## Measure the Earth Mover’s Distance (EMD)*

     phi = D(partitioned_purview || unpartitioned_purview)

    phi_cause_max = max(phi)

   for each element in the future purview:

    *## Calculate phi_effect_max*

    for every partition of the purview element:

     *## Measure the Earth Mover’s Distance (EMD)*

     phi = D(partitioned_purview || unpartitioned_purview)

    phi_effect_max = max(phi_effect_mip)

   phi_max = min(phi_cause_max,phi_effect_max)

  *## Measure the extended EMD*

  Phi = D(original_ces || new_ces)

 Phi_MIP = min(Phi)

Phi_Max = max(Phi_MIP)

Specifically, it is the calculations of $\phi^{Max}_{cause}$ and $\phi^{Max}_{effect}$ in Lines 12 and 17 of Algorithm 1 that are the source of non-uniqueness in the routine for calculating Φ. In both cases, the problem amounts to an inability to uniquely specify the purview element associated with the minimum *ϕ*^*MIP*^ value, as shown in Figure A2. In Section “Mathematical Details”, we provide a detailed explanation of this problem, but the key feature is that both the minimum *ϕ* value and the probability distribution associated with the corresponding purview element are carried forward in the calculation. If it were just the scalar *ϕ* value that was carried forward, then there would be no meaningful difference between degenerate values. However, in Line 19 of Algorithm 1, we must calculate a distance between probability distributions, called cause-effect structures (CESs), and the shape of these distributions depends on which purview element is associated with the minimum *ϕ* value. In other words, the purview element with the minimum *ϕ* value determines the shape of the probability distributions going into the calculation of Φ, and these distributions can have different shapes with the same minimum *ϕ* values.

To address this problem, one needs to consider each of the tied purview elements in turn. The Python package PyPhi (available for download at http://integratedinformationtheory.org) provides all the basic functionality needed to do this. Namely, it allows the user to calculate purviews, cause/effect repertoires, Earth Mover’s Distance (EMD) Pele and Werman (2008), CES distance (also known as the extended EMD), and more. In addition, it contains prebuilt classes for data structures corresponding to concepts that are useful in the calculation. Thus, our methodology is to wrap the basic functionality of PyPhi into a modified version, which we call PyPhi-Spectrum, that allows the user to calculate all possible Φ values for a given subsystem using the prebuilt PyPhi functions. Our algorithm follows the mathematical definition of IIT precisely, with the only difference to PyPhi being that whenever there is a minimization or maximization procedure, it looks for all minimizers or maximizers, forks the state of the computation accordingly, and carries on computations according to the mathematical definition for all forks (Section “The PyPhi-Spectrum package”). To install PyPhi Spectrum, download or clone the repository from https://github.com/elife-asu/pyphi-spectrum.

As a concrete example of our methodology, we consider the simple AND+OR system shown in Figure 1(a). Here, each cut generates a host of different Φ values due to the degeneracy in the optimization routine, as discussed in detail in Section “Mathematical Details”. Feeding this system into PyPhi-Spectrum, we find that there are 83 valid $\Phi^{MIP}$ values, as opposed to the single $\Phi^{MIP}$ value output from PyPhi. Crucially, both $\Phi=0$ and $\Phi\gt0$ are predicted, meaning that there is no longer a clear prediction as to whether or not this system is conscious.

## Results

In this section, we apply PyPhi-Spectrum to a variety of recently published systems in order to determine the extent to which non-uniqueness affects the interpretation of results in the published literature.

### Case study: three-node fission yeast cell cycle

**Figure 2.**
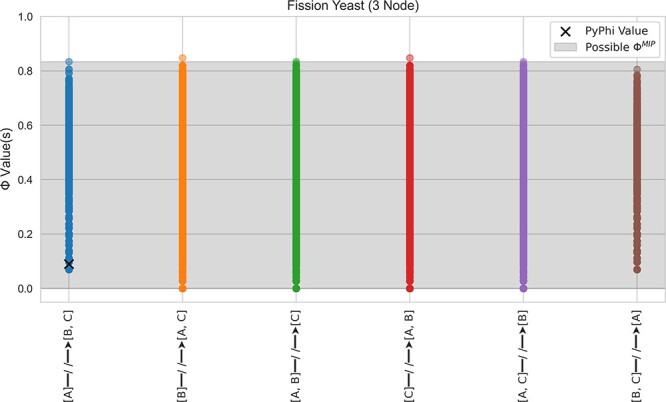
The spectrum of Φ values that result from consideration of degenerate core causes/effects in the three-node fission yeast system analyzed by Marshall et al. (2017). The published value for this system is shown as an “x”, but all Φ values are equally valid according to the mathematical definition of Φ. The shaded region contains the set of all valid $\Phi^{MIP}$.

As a case study, we first consider the Boolean network model of the fission yeast cell cycle from Marshall et al. (2017) (one of our co-authors was also an author on this study). In the study by Marshall et al., IIT is used to analyze the causal structure of a minimal biological system, namely, the cell cycle of the fission yeast *Schizosaccharomyces pombe*. Using Φ values output from PyPhi, the authors identify three integrated subsystems corresponding to the full regulatory network (eight nodes), a six-node subsystem, and a three-node subsystem—all potentially of biological importance. Of these three systems, only the smallest may be subject to our analysis, although we expect similar results for the other two systems. Applying the methodology from Section “Methodology”, we find a spectrum of 244 non-unique Φ values, spanning a range from 0.00 to 0.83 bits, as shown in Figure 2. Only one of these values ($\Phi=0.09$) is published as the unique Φ value for this subsystem. Furthermore, the inclusion of $\Phi=0$ in the spectrum of possibilities changes the biological narrative of the results entirely. If the subsystem under consideration has $\Phi=0$, rather than $\Phi\gt0$, it would not be identified as “integrated” and the connection between integration and the autonomy discussed in the manuscript cannot be made. Thus, the conclusion that this subsystem is of biological importance is entirely dependent on the arbitrary selection of a single Φ value from the spectrum of possibilities and, in general, it is impossible to tell *a priori* whether the interpretation of results from a singular Φ value is valid without considering the entire spectrum of possibilities.

### The non-uniqueness of published Φ values

Next, we use PyPhi-Spectrum to analyze a corpus of recently published Φ values. This corpus is meant to be comprehensive but is not exhaustive, in particular due to the limitations imposed by the computational resources required to perform these calculations (Section “On the computational complexity of Φ”). Of the dozen or so Φ values that are published in the literature Haun and Tononi (2019); Albantakis et al. (2019); Albantakis and Tononi (2019); Juel et al. (2019); Popiel et al. (2020); Hanson and Walker (2019); Hanson and Walker (2021); Nilsen et al. (2019); Aguilera et al. (2018); Marshall et al. (2017); Farnsworth (2021); Niizato et al. (2020); Tononi et al. (2016); Hoel et al. (2016); Chalmers and McQueen (2014), only a handful are small enough to be subjected to our analysis Albantakis et al. (2019); Hanson and Walker (2021); Farnsworth (2021); Marshall et al. (2017); Oizumi et al. (2014); Tononi et al. (2016); Hoel et al. (2016); Chalmers and McQueen (2014). These systems, summarized in Table 1, are selected primarily for their size, although size alone is not a good indicator of computational tractability. For example, certain three-node systems, such as that of Hanson and Walker (2021), have thousands of degenerate CESs, while other three-node systems, such as that of Farnsworth (2021), have just a few. It is not readily apparent what dictates the number of non-unique CESs that, in turn, govern tractability, although underlying symmetries undoubtedly play a role. Consequently, our corpus is characterized by small systems (2–4 nodes), which allow relatively fast evaluation via PyPhi-Spectrum^2^.

**Table 1.** Summary of the corpus in reverse chronological order. Sources were selected based on the publication of a Φ value and computational tractability. Additional details required for analysis, such as transition probability matrices and initial states, are provided in Section “Additional details related to the calculation of Φ values” (Table A1–A11).

Name	Description	Size	Φ value	Ref(s)
AND+OR	System from Section “Methodology”	2	0.0903	Hanson and Walker (2019); Albantakis et al. (2019)
Farnsworth 2021 (full)	Virus–host dynamics	5	0.3125	Farnsworth (2021)
Farnsworth 2021 (reduced)	Simplified virus–host dynamics	3	0.4375	Farnsworth (2021)
Gomez et al. 2020	p53-Mdm2 regulatory network	4	0.2153	Gomez et al. (2021)
Photodiode	COPY+COPY	2	1.0000	Chalmers and McQueen (2014); Oizumi et al. (2014)
Hanson and Walker 2020	Mod-eight digital counter	3	1.7187	Hanson and Walker (2021)
Marshall et al. 2017	Fission yeast cell cycle	3	0.0903	Marshall et al. (2017)
Hoel et al. 2016	AND+AND+AND+AND	4	0.1139	Hoel et al. (2016)
Tononi et al. 2016	MAJORITY+OR+AND+AND	4	0.6597	Tononi et al. (2016)
Oizumi et al. 2014	OR+AND+XOR	3	1.9167	Oizumi et al. (2014)

**Figure 3.**
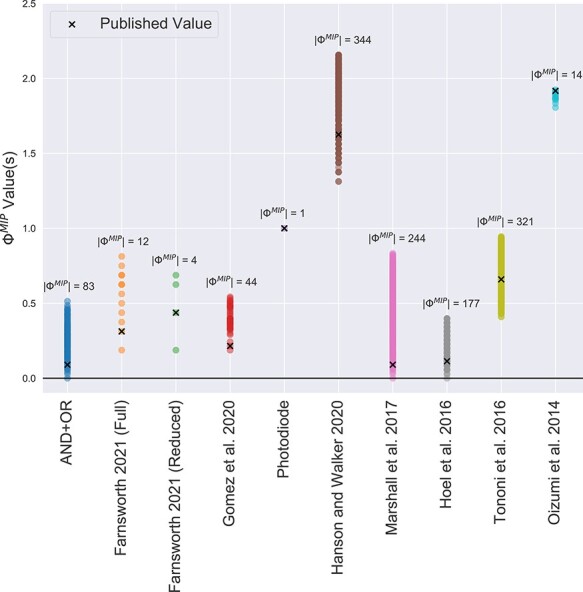
Possible Φ values relative to the published value for the corpus shown in Table 1. Each point represents a possible value that results from the mathematical definition of Φ. The number of different values for each system is given by the cardinality of its spectrum $|\Phi^{MIP}|$.

Our primary result is shown in Figure 3 and demonstrates the entire spectrum of possible Φ values for each system in our corpus relative to its published value. There are several things to note. First, the existence of a unique Φ value is rare: only the photodiode example has a spectrum consisting of a single value (the number of different $\Phi^{MIP}$ values is denoted by $|\Phi^{MIP}|$ in Figure 3). For the rest of the corpus, the spectra often consist of dozens if not hundreds of non-unique values, of which only one is published (denoted as an “*x*” in Figure 3). There are also multiple cases where the spectrum contains both $\Phi=0$ and $\Phi\gt0$ values, demonstrating how in its current formal implementation, IIT is unable to predict whether or not such systems are conscious when non-uniqueness is properly accounted for in the definition of Φ. This occurs for 3 out of the 10 published Φ values in our corpus: AND+OR, Marshall et al. (2017), and Hoel et al. (2016). This has implications for the logical foundations of the theory and, in particular, for the exclusion postulate, as this postulate causes the degeneracy (Section “Conclusion”). Additionally, the span of possible Φ values is often comparable to the entire range of possibilities expected for systems of this size. Typical Φ spectra in Figure 3 span roughly half of a bit for two node Boolean systems, which are bound from above by $\Phi^{MIP} \leq 1.5$ and from below by $\Phi^{MIP}=0$ (c.f. Section “Calculating an upper bound on Φ”). This implies that the Φ values specified by IIT are not only non-unique but also non-specific in the sense that they do not constrain the Φ value to a small portion of the possible range.

## Discussion

### Implementing existing solutions

**Figure 4.**
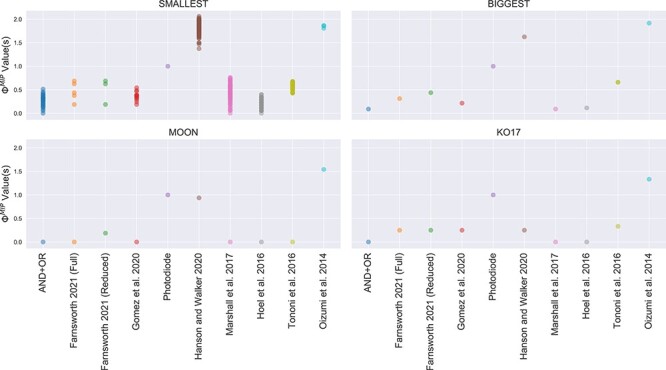
The spectrum of Φ values that results for our corpus using each of the four proposed solutions. The “smallest” and “biggest” solutions do not guarantee a unique Φ value, while the Moon and KO17 solutions result in $\Phi=0$ for systems that are clearly integrated, such as the AND+OR gate.

**Figure 5.**
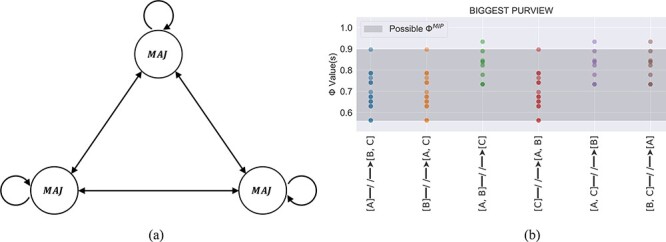
For a system of three fully connected majority gates, (a) selecting the largest purview element does little to mitigate degeneracy, as evident by the range of possible $\Phi^{MIP}$ values (b).

In Section “The non-uniqueness problem”, we discussed logical problems inherent with each of the four proposed solutions to the non-uniqueness problem. Even so, we can easily apply these solutions to our corpus using PyPhi-Spectrum^3^ and study the results, shown in Figure 4. As expected, the KO17 and Moon solution yield $\Phi=0$ for several systems that are clearly integrated, such as the AND+OR system. Indeed, the fact that the Moon solution yields $\Phi=0$ for so many systems that were previously considered integrated suggests that Moon’s solution deviates from the accepted notion of an integrated system in IIT. It is also clear that the “smallest” criterion does little to mitigate the degeneracy. At first glance, it appears that the “biggest” criterion avoids both these issues and provides a unique positive Φ value in all cases. However, this is an idiosyncrasy of our dataset and, in general, selecting the biggest purview element will result in the same problem as selecting the smallest purview element; namely, degenerate core causes/effects are often the same size. The reason that the “biggest” solution appears to yield unique Φ values for the systems under consideration is due entirely to the over-representation of two-input logic gates (AND, OR, XOR, NOR, etc.) in our corpus. In such cases, the tied purview elements are almost always *A*, *B*, and *AB*, so selecting the largest purview element results in the unique core cause/effect *AB*. However, this does not hold generally as systems comprised of logic gates with more than two inputs (such as neurons in the human brain) have entirely different symmetries. As Figure 5 shows, a simple system comprised of majority gates with three inputs each is enough to prove that a unique Φ value is far from guaranteed with the “biggest” criterion and the problem remains fundamentally unaddressed.

### On the scaling of non-uniqueness with the system size

It is easy to assume that the non-uniqueness problem is irrelevant for systems as large as the human brain. The intuition behind this argument is that as the system size grows, the chance of two different distributions yielding the same *ϕ* value decreases due to the increased size of the underlying support. However, as the system size increases so too does the number of cause/effect repertoires under consideration. So, on the one hand, the likelihood of any individual pair of elements being tied diminishes, but on the other hand, there are exponentially more combinations of elements (and partitions of those combinations) that must be considered. A detailed analysis must be performed in order to assess these competing factors.

One approach is to calculate the number of possible *ϕ* values—denoted $|\phi|$—as a function of the system size *n*. We then compare this to the number of purview elements under consideration, which is always $n!$ in order to determine if degenerate *ϕ* values are guaranteed by the pigeonhole principle Herstein (1991). In short, if there are more *ϕ* values than there are purview elements, then there must be degenerate purview elements. We introduce a measure of degeneracy $L = \frac{n!}{|\phi|}$ that can be used to track how many degenerate *ϕ* values are assigned to each purview element on average. The scaling of *L* as a function of *n* determines whether or not degeneracy plays a role in larger systems.

For example, let us say that probability distributions come in discretized units of $1/n$, where *n* is the number of states in the system; note that this is the case for all deterministic transition probability matrices. The number of unique probability distributions that can be built subject to this constraint is equivalent to the number of ways we can assign *n* units of probability to *n* different states (the distribution’s support). For each of the *n* units, there are *n* choices, so the number of unique probability distributions is at most *n*^2^. Since *ϕ* is a distance measure between two probability distributions, the number of unique *ϕ* values is at most $|\phi| = n^2 \times n^2$. If we then compare this to the number of purview elements under consideration, we find $L = \frac{n!}{n^4}$, which goes to infinity as *n* goes to infinity. This implies that degeneracy gets worse, and not better, with increasing system size.

## Conclusion

In addition to the mathematical problem of non-uniqueness, there is a philosophical problem as well. Namely, non-uniqueness arises as a direct consequence of the exclusion postulate in IIT, which is used to localize consciousness into singular experiences. Mechanistically, the way this is achieved is by suppressing the multitude of intermediate Φ values in favor of the extremum, which can then be uniquely mapped onto a single conscious percept. Non-uniqueness, then, implies a philosophical problem in which there is no clear way to map the multitude of Φ values onto a single conscious percept—either all the Φ values are valid, which results in simultaneous conscious experience, or an additional postulate is required to justify the choice between values. In the former case, it is a phenomenological axiom that is violated, while the latter case requires at least one additional postulate. In both cases, the revision goes well beyond a simple mathematical fix and points to problems with the so-called “hard core” of the theory Negro (2020); Lakatos (1968). It is for this reason that Moon emphatically argues “the underdetermination problem shakes IIT to the ground” Moon (2019).

The problem of non-uniqueness in IIT fits into a more general problem faced by phenomenology-first theories of consciousness. If we begin with a small set of axioms, it is virtually impossible for these axioms to uniquely constrain a precise mathematical measure. At each step in the operationalization, we must add details to the phenomenology in order to define the mathematics, which means that either the mathematical choices lack phenomenological grounding or the list of phenomenological axioms becomes increasingly contrived. For example, in IIT 2.0, the Kullback–Leibler divergence was used to calculate distances between probability distributions, whereas the EMD was used in IIT 3.0. Mathematically, these two measures are very different and will yield qualitatively different predictions for the same system. This implies that only one of the two measures can be correct, yet it is difficult to imagine how one can justify the choice between these two distance measures in terms of “what it is like” to be conscious Nagel (1974). If we are to take a phenomenology-first approach to consciousness, we must accept that there is likely not going to be a unique measure of consciousness that results from the phenomenology of the theory, but rather, a host of different measures that are *consistent* with the axioms of the theory. We must then rely on experiments, grounded by independent benchmarks, to falsify the different quantitative predictions.

To be clear, the non-uniqueness problem in IIT 3.0 is not a case of a precise mathematical measure that fails to find justification in terms of its phenomenological axioms, but rather, an ill-defined mathematical measure that demonstrably violates the hard core of the theory. That said, any proposed solution to the non-uniqueness problem must contend with the general lack of specificity provided by a phenomenology-first approach. Such an approach requires experimental evidence as a means to justify the specific choices that were made in translating phenomenological axioms into precise mathematics. There is a growing body of literature discussing how experiments may be used to assess different theories of consciousness Doerig et al. (2020) and how the mathematical structure of the theory, in general, must be consistent with what is known experimentally about conscious percepts Kleiner and Ludwig (2023). Assessing the impact of non-uniqueness, in all of its forms, is a crucial part of this research paradigm.

## Appendix

### Mathematical details

To demonstrate the problems inherent in the mathematical definition of Φ, we will consider a simple system comprising an AND gate and an OR gate connected to each other, as shown in Figure 1. Since there are only two elements, we need not worry about the outermost optimization over subsystems as a two-element system can have only one meaningful subsystem ( i.e. $\Phi^{MIP}=\Phi^{Max}$ for two element systems). To calculate $\Phi^{MIP}$, we first must initialize the system into a given state. Here, we assume an initial state $s_0 = 00$, with the understanding that this specific choice of initial state does not impact the general result. The next step is to identify the CES or constellation *C* corresponding to the transition probability matrix (TPM) of the unpartitioned system. To do this, one must find the “core cause” and “core effect” of every potential mechanism in the system, where a mechanism is any element in the power set of the subsystem. In this case, the potential mechanisms are in $\mathcal{P}(\{A^cB^c\})=\{A^c,B^c,AB^c\}$, where the superscript *c* denotes the mechanism in its current state. For each element in this set, we must identify how well it constrains elements in the past power set $\mathcal{P}(\{A^pB^p\})$, known as the past purview, as well as how well it constrains elements in the future powerset $\mathcal{P}(\{A^fB^f\})$, known as the future purview.

**Figure A1.**
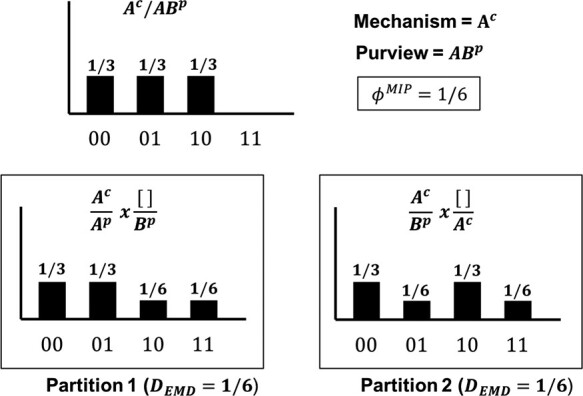
All possible partitions of a given mechanism and purview combination and the resulting *ϕ*^*MIP*^ value.

We then measure the EMD Pele and Werman (2008)  *D* between the constrained distribution of each purview element and the constrained distribution of each purview element under the “minimum information partition” (MIP):


$$ \phi^{MIP}(m,z) = D[p(z|m=s_0)||p(z|m=s_0/\textrm{MIP})], $$

where *z* is the purview element and *m* is the mechanism. The distribution $p(z|m=s_0)$ tells us the likelihood of *z* given that the current state of *m* is *s*_0_, which, compared to an unconstrained distribution, tells us how much information *m* is generating about *z*. We also need to know whether or not that information is integrated, so we must break *m* and *z* up into all possible parts and ask whether the parts acting independently can generate the same amount of information as the whole. For example, to find how much integrated information is generated by the mechanism *A*^*c*^ about the purview element $z = AB^p$, we calculate the probability distribution $p(AB^p|A^c = 0)$ and compare this to the two possible partitions of the purview: $A^c/AB^p \rightarrow (A^c/A^p \times [ ]/B^p)$ and $A^c/AB^p \rightarrow (A^c/B^p \times [ ]/A^p)$. The first partition allows *A*^*c*^ to constrain *A*^*p*^ but leaves *B*^*p*^ unconstrained (denoted by an empty bracket $[ ]$), while the second partition allows *A*^*c*^ to constrain *B*^*p*^ but leaves *A*^*p*^ unconstrained. The distributions generated by these partitions, shown in Figure A1, are then compared to the distribution generated by the unpartitioned system, and the partition that minimizes the EMD to the unpartitioned system is the MIP for this purview/mechanism combination. If multiple partitions yield the same distance to the unpartitioned system, as is the case in Figure A1, it is irrelevant which one is chosen as all that moves forward from this step of the computation is the scalar value of *ϕ*^*MIP*^.

**Figure A2.**
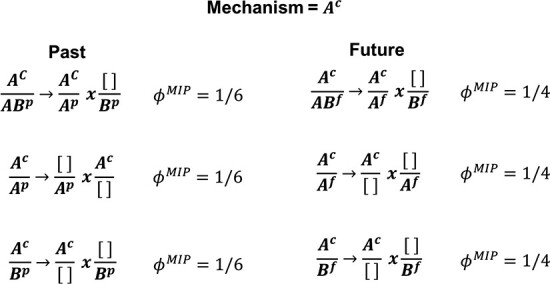
All possible purview elements and their MIPs for a given mechanism. It is here that the degeneracy is introduced, as one cannot select a unique core cause or effect for a given mechanism if there are purview elements with the same *ϕ*^*MIP*^ values.

Once we have identified the MIP (and calculated *ϕ*^*MIP*^) for all purview elements for a given mechanism, we define the core cause and core effect as the past and future purview elements with the greatest *ϕ*^*MIP*^. We denote the integrated information of the former as $\phi_{cause}^{Max}$ and of the latter as $\phi_{effect}^{Max}$ and define the total integrated information *ϕ*^*Max*^ of a given mechanism as


$$ \phi^{Max} = \min{[\phi_{cause}^{Max},\phi_{effect}^{Max}]}. $$

If $\phi^{Max}\gt0$ for a mechanism we say that the mechanism gives rise to a “concept”. A concept is a tuple comprising of three things: a scalar *ϕ*^*max*^ value, a probability distribution corresponding to the core cause repertoire, and the probability distribution corresponding to the core effect repertoire. We have already provided an example of a cause repertoire in Figure A1, namely, it is the distribution over previous states of the purview element given the current state of the mechanism. In Figure A1, the state of *A*^*c*^ constrains the probability of observing *AB*^*p*^ and this constrained distribution is the cause repertoire for the purview element *AB*^*p*^. Any element not included as part of the purview is left unconstrained and must be independently “noised” Oizumi et al. (2014). For example, if the purview of mechanism *A*^*c*^ is *A*^*p*^, we generate the constrained distribution $p(A^p|A^c=s_0)$ and combine this with the unconstrained distribution for *B*^*p*^ (denoted $p^{uc}(B^p)$). For the AND+OR system, we have $p(A^p|A^c=0) = [2/3,1/3]$ and $p^{uc}(B^p) = [1/2,1/2]$, which yields the cause repertoire: $[1/3, 1/3, 1/6, 1/6]$, where states are ordered in binary with the most significant digit on the left ($00,01,10,11$).

The effect repertoire for a given purview element is generated in the same way as a cause repertoire. For example, the probability of observing *A*^*f*^ given the state of mechanism $A^c =0$ is $P(A^f|A^c=0) = [0,1]$. Combining this with the unconstrained future distribution for *B*^*f*^ yields the effect repertoire $[1/4, 3/4, 0, 0]$. Note that this is an example where the unconstrained future distribution for *B*^*f*^ is not uniform. This is a direct result of the noising procedure: an OR gate receiving uniform random input is three times as likely to be in State 1 as it is to be in State 0. Furthermore, when more than one target node is involved, we must send independent noise to each target (to avoid correlated input).

We can now define the CES (constellation) *C* as the set of all concepts for the system in the given state. Recall that each concept corresponds to a single mechanism and comprises the mechanism’s core cause repertoire, core effect repertoire, and *ϕ*^*Max*^ value. In our example, there are at most three concepts, corresponding to the mechanisms $\{A^c, B^c, AB^c\}$. The core cause repertoire for a mechanism is found by optimizing over all past purview elements and identifying the purview with the highest *ϕ*^*MIP*^, where *ϕ*^*MIP*^ is found by further optimization over all possible partitions. Figure A2 shows *ϕ*^*MIP*^ and the corresponding partition for all possible purview elements given the mechanism *A*^*c*^.

#### Degenerate core causes and effects

We are now in a position to see how degenerate core causes and effects can occur and their consequences. The postulates of IIT—and the exclusion postulate, in particular—imply that a unique core cause should be assigned to each mechanism, but the purview element that generates $\phi_{cause}^{Max}$ is not unique. As Figure A2 shows, *A*^*p*^, *B*^*p*^, and *AB*^*p*^ all generate the same *ϕ*^*MIP*^ value for the mechanism in question. Since each purview/mechanism combination is associated with a different cause repertoire, “the core cause repertoire and the resulting constellation *C* are not uniquely defined”. If the scalar value of $\phi^{Max}_{cause}$ was all that mattered to the calculation of Φ, this degeneracy would be inconsequential (as is the case for partitions that generate the same *ϕ*^*MIP*^ value for a given purview element). However, system-level integrated information Φ is defined as the cost of transforming the core cause/effect repertoires from one constellation *C* into another *C*^ʹ^. That is,


$$ \Phi = D(C||C^{\prime}), $$

where *D* is an extension of the EMD that calculates the cost of moving *ϕ*^*Max*^ “between repertoires” Oizumi et al. (2014). If the core cause or effect repertoire changes, the distance between constellations will change accordingly, as the distance metric that goes into the EMD calculation is sensitive to the relative shape of the distributions and not just the scalar *ϕ*^*Max*^ values. For example, if we were to choose *AB*^*p*^ as the core cause for mechanism *A*^*c*^, this generates the concept in *C* given by the tuple $\{[1/3, 1/3, 1/3, 0], [1/2, 1/2, 0, 0],1/6\}$, where the first element is the core cause repertoire, the second element is the core effect repertoire, and the third element is the *ϕ*^*Max*^ value. However, we could just as easily have chosen *A*^*p*^ as our core cause and *A*^*f*^ as our core effect. In which case, the concept generated for *A*^*c*^ would be $\{[1/3, 1/3, 1/6, 1/6], [1/4, 3/4, 0, 0],1/6\}$. Clearly, these choices have the same *ϕ*^*Max*^ value ($1/6$) but significantly different cause/effect repertoires.

**Algorithm 2:** Basic usage for the PyPhi-Spectrum Wrapper

import pyphi

import numpy as np

from pyphi import phi_spectrum

 

*# Transition probability matrix.*

tpm = np.array([

 [0.,0.,0.],

 [0.,0.,0.],

 [1.,0.,0.],

 [1.,0.,1.],

 [0.,1.,0.],

 [0.,1.,0.],

 [1.,1.,0.],

 [1.,1.,1.]

])

 

*# Set up network object*

network = pyphi.Network(tpm, node_labels=[‘A’,‘B’,‘C’])

print(“Network = ”,network.node_labels)

 

*# Put the system into a given state*

state = (0,0,0)

nodes = [‘A’,‘B’,‘C’]

 

*## Get the requisite Subsystem*

subsystem = pyphi.Subsystem(network, state, nodes)

 

*## Calculate all Phi values*

display_CES = False *# if True, output will display constellations*

solution = None *# how to handle degenerate purview elements (‘Smallest’,‘Largest’, or ‘Moon’)*

Phi_Spectrum = phi_spectrum.get_phi_spectrum(subsystem,display_CES,solution)

 

print(“nCuts = ”,Phi_Spectrum[0])

print(“nPhi Spectrum = ”,Phi_Spectrum[1])

 

Phi_MIP = phi_spectrum.get_Phi_MIP(Phi_Spectrum)

print(“Phi MIP = ”,Phi_MIP)

**Algorithm 3:** Pseudocode overview of the get_Phi_spectrum function

*## Return the spectrum of Phi values*

def get_Phi_spectrum(subsystem):

 

  *## Initialize an empty list to store all Phi values for all cuts*

  Phi_Spectrum = []

  *## Find all concepts for the specified subsystem*

  all_concepts = get_all_concepts(subsystem)

  *## Create all possible cause-effect structures (CES)*

  original_CES = get_all_CES(all_concepts)

  print(”tNumber of Non-unique Constellations =“,len(original_CES))

   

  *## Now cut the original TPM and find the new set of concepts. Get the Phi value and repeat.*

  bipartitions = get_all_bipartitions(subsystem.cut_indices, subsystem.cut_node_labels)

  for cut in bipartitions:

    print(“nEvaluating Cut ”,cut)

    new_subsystem = subsystem.apply_cut(cut)

     

    *## Find all concepts for the specified subsystem*

    new_concepts = get_all_concepts(new_subsystem)

    new_CES = get_all_CES(new_concepts)

     

    print(”tNumber of Non-unique Constellations =“,len(new_CES))

     

    *## Now store all possible Phi values for this cut*

    Phi_cut = []

    for original in original_CES:

     for new in new_CES:

      Phi = ces_distance(original, new)

      if Phi not in Phi_cut:

        Phi_cut.append(Phi)

    *## Append the list of Phi values to the spectrum*

    Phi_Spectrum.append(Phi_cut)

 

  return(bipartitions,Phi_Spectrum)

**Algorithm 4:** Pseudocode overview of the get_Phi_MIP function

*## Return all Phi values between the min and max Phi value of the MIP*

def get_Phi_MIP(phi_spectrum):

 

 cuts = phi_spectrum[0]

 values = phi_spectrum[1]

 

 *## Get the minimum Phi value of the MIP*

 Phi_min_MIP = min(values[0])

 for each in values:

  if min(each) < Phi_min_MIP:

   Phi_min_MIP = min(each)

 

 *## Get the of MIP(s) corresponding to the minimum Phi value*

 possible_MIPs = (index for index in values if min(index) == Phi_min_MIP)

 

 *## Upper bound on Phi is the smallest of the max Phi values from the possible MIPs*

 Phi_max_MIP = max(values[0])

 for each in possible_MIPs:

  if max(each) < Phi_max_MIP:

   Phi_max_MIP = max(each)

*## Return all Phi values between Phi_min_MIP and Phi_max_MIP*

 valid_phi = [phi for index in values for phi in index if phi >= Phi_min_MIP and phi <= Phi_max_MIP]

 return(unique(valid_phi))

To illustrate the consequences of this, let *C* be the constellation consisting only of the concept generated by *A*^*c*^ with core cause *AB*^*p*^ and core effect *AB*^*f*^ and let *C*^ʹ^ be the constellation consisting of only the null concept for this system (the unconstrained cause and effect repertoires):


$$ C = \{[1/3, 1/3, 1/3, 0], [1/2, 1/2, 0, 0],1/6\}, $$

$$ C^{\prime}= \{[1/4, 1/4, 1/4, 1/4], [3/16, 9/16, 1/16, 3/16],0\}. $$

The extended EMD is the cost of transforming *C* into *C*^ʹ^ by moving $\phi^{Max}=1/6$ a distance given by the sum of the (regular) EMD between cause repertoires and effect repertoires. Namely, we have


$$ D_{cause} = D_{EMD}([1/3,1/3,1/3,0]||[1/4,1/4,1/4,1/4])=1/3, $$

$$ D_{effect} = D_{EMD}([1/2,1/2,0,0]||[3/16,9/16,1/16,3/16])=1/2, $$

which results in the integrated conceptual information,


$$ \Phi = (D_{cause}+D_{effect})\phi^{Max}=(\frac{1}{3}+\frac{1}{2})\frac{1}{6}=\frac{5}{36}. $$

Now, if we instead choose *A*^*p*^ and *A*^*f*^ as our core cause and core effect, we have


$$ C = \{[1/3, 1/3, 1/6, 1/6], [1/4, 3/4, 0, 0],1/6\}, $$

$$ D_{cause} = D_{EMD}([1/3,1/3,1/6,1/6]||[1/4,1/4,1/4,1/4])=1/6, $$

$$ D_{effect} = D_{EMD}([1/4,3/4,0,0]||[3/16,9/16,1/16,3/16])=1/4, $$

corresponding to an integrated conceptual information,


$$ \Phi= (\frac{1}{6}+\frac{1}{4})\frac{1}{6}=\frac{5}{72}. $$

Thus, we get different values of Φ depending on our choice of core cause and core effect. In total, 83 different Φ values result from this process, as shown in Figure 1(b).

The last step is to calculate $\Phi^{MIP}$, which is defined as the minimum Φ value over all possible cuts. For the AND+OR system, there are only two possible cuts; however, each cut has a host of possible Φ values. We must consider the pairwise combination of the Φ values from each cut in turn and ask what the minimum is in order to build up the set of possible $\Phi^{MIP}$ values. For example, if the Φ values for cut 1 are $[0,1,2]$ and the Φ values for cut 2 are $[1,2,3]$, then only $\Phi=3$ is excluded from being a valid $\Phi^{MIP}$ value. The reason for this is that all other Φ values are the minimum of some combination of Φ values from the cuts under consideration (e.g. when $\Phi=3$ in cut 2 and $\Phi=2$ in cut 1, then $\Phi^{MIP}=2$), but there is no situation for which $\Phi=3$ is the minimum across all cuts because the maximum Φ value from cut 2 is always less than $\Phi=3$. In general, the maximum $\Phi^{MIP}$ value is set by the cut with the smallest $\max{\Phi}$ value, as evident in Figure 5(b).

### On the computational complexity of Φ

For a subsystem of size *n*, the computational complexity scales as follows. First, one must calculate the CES for every possible partition of the subsystem. If the partition is a bipartition, as is typically assumed Mayner et al. (2018); Krohn and Ostwald (2017), the number of ways to do this is $S(n,2)$, where *n* is the size of the subsystem and $S(n,m)$ are Stirling numbers of the second kind Stanley (2011). However, two small modifications must be applied: first, partitions are unidirectional, and second, the unpartitioned system must also be included. The former consideration results in twice as many bipartitions, while the latter results in a single additional partition. Combining these results in a total of $2S(n,2)+1$ partitions, which, for large *n*, is well approximated as $2S(n,2)$. For each CES, there are $2^n-1$ potential mechanisms, corresponding to the size of the powerset of elements excluding the empty set. For each potential mechanism, there are $\binom{n}{k}$ purview elements of size *k*, each of which can be partitioned $S(k,2)$ times. Therefore, there are $2\sum_k \binom{n}{k}S(k,2) = 2(3^n)$ elementary distance calculations that must be performed to calculate a single CES, where the additional factor of two is due to the need to optimize *ϕ*^*max*^ over both past and future purviews. Putting this together, there are a total of $2S(n,2+1) \times (2^n-1)\times 2(3^n) \approx 12^n$ elementary distance calculations required to get the system-level integrated information $\Phi^{MIP}$ for a given subsystem. For the global system, this calculation must be embedded in an additional optimization corresponding to maximizing over the powerset of all possible subsystems (i.e. $\Phi^{max} = \textrm{max}\{\Phi^{MIP}\}$). For a global system of size *m*, there are $\binom{m}{n}$ subsystems of size *n*, each with 12^*n*^ elementary distance calculations. Therefore, there are a total of $\sum_n \binom{m}{n} 12^n = 13^m$ elementary calculations required to find $\Phi^{max}$ for a global system of size *m*. For all but the smallest *m* values, the computational resources required to actually calculate $\Phi^{max}$ are impossible to realize.

Interestingly, the $\mathcal{O}(13^m)$ scaling derived here is in tension with the previously published value of $\mathcal{O}(53^m)$  Mayner et al. (2018). This could be due to the possibility that the $\mathcal{O}(53^m)$ scaling considers all possible partitions, rather than strict bipartitions, or perhaps it resolves the elementary computation in terms of some more fundamental operation (e.g. bit flips). Without additional information on how $\mathcal{O}(53^m)$ was derived, it is difficult to say whether either of these considerations resolve the tension between values. We do note, however, that the published values of *t* = 1, *t* = 16, and $t=9900s$ for *n* = 3, *n* = 5, and *n* = 7 (Mayner et al. (2018)) are within an order of magnitude of the calculated $\mathcal{O}(13^m)$ scaling but off by 40 orders of magnitude from $\mathcal{O}(53^m)$ scaling.

### Calculating an upper bound on Φ

It is relatively straightforward to calculate a loose upper bound on Φ for a subsystem of size *n*. To do so, one needs to only understand the extension of the earth mover’s distance *D* that is used in the calculation $\Phi=D(C||C_{\rightarrow})$. By definition, the “earth” being moved is *ϕ*^*Max*^ between concepts in the unpartitioned CES (C) and the partitioned CES ($C_{\rightarrow}$), while the “distance” it is moved is measured by the regular earth mover’s distance between concepts Oizumi et al. (2014). In light of this, a straightforward upper bound on Φ can be found by asking what the maximum value of *ϕ*^*Max*^ is for each concept and moving that amount as far away as possible. For a mechanism of size *m*, its *ϕ*^*Max*^ value is bound from above by the maximum value of the regular earth mover’s distance, which is $EMD^{Max}(m) = m$. It is easy to see this is the case, as EMD$^{\rm Max}$ is achieved when all the probability (*P* = 1) is moved the maximum Hamming distance (*H*^*Max*^), which is *m* for a mechanism comprising *m* bits. For example, *EMD*^*Max*^ for a three-bit mechanism is achieved when *P* = 1 is moved from State 000 to State 111 ($H^{Max}=3$), so $\phi^{max} = EMD^{Max} = 3$. Next, we must ask what the maximum distance *D*^*Max*^ in conceptual space is that this amount of *ϕ*^*Max*^ can be moved. Since this distance is again a regular earth mover’s distance, we have $D^{Max} = EMD^{Max}(m) = m$. Thus, the maximum contribution a mechanism of size *m* can make to the extended earth mover’s distance *D* is upper bound by $\phi^{Max}(m)D^{Max}(m) = [EMD^{Max}(m)]^2 = m^2$. Of course, not all mechanisms are the same size, so the total contribution is bound by the sum of the maximum contribution from mechanisms of each size, namely,


$$ \Phi(n) \leq \sum_{m=1}^{n} \binom{n}{m}m^2 = 2^{n-2}n(n+1). $$

To date, this is the only known upper bound on Φ that we are aware of (although bounds on *ϕ*^*max*^ and in IIT 2.0 are readily available Krohn and Ostwald (2017); Oizumi et al. (2016); Arsiwalla and Verschure (2016); Tegmark (2016); Toker and Sommer (2016)), and it is a rather loose bound. For a subsystem of size *n* = 2, as is the case for the AND+OR system, we consider in the main text that we have $\Phi(2) \leq (1)^2 + (1)^2 + (2)^2 =6$ bits. In practice, we cannot reasonably expect $\phi^{Max} = EMD^{Max}$ for all mechanisms, as the existence of $\phi^{Max}=EMD^{max}$ for one mechanism almost certainly precludes the existence of $\phi^{Max} = EMD^{Max}$ for another. Likewise, cutting a CES cannot possibly result in a distance of $D^{Max} = EMD^{Max}$ for all concepts, as additional noise cannot be used to increase the fidelity of constraints. At best, it is likely that concepts map to the null concept in the *CES* of the MIP, corresponding to a maximum distance $D^{Max}(n) = n/2$. In this case, the bound that results is $\Phi \leq2^{n-3}n(n+1)$, which is still likely loose. To tighten it, one must consider the *ϕ*^*Max*^ values that can result for a system of mechanisms as an ensemble, rather than individually, which is a task that we found quickly became intractable.

#### Numerical approach

For our purposes, a numerical approach will suffice. Given a small enough system, it is possible to calculate the Φ values for every possible TPM that result from Boolean logic on a two-bit system. Namely, each bit (*A* and *B*) takes one of two possible states in response to the global state of the system. This means that there are $2^4=16$ possible state transitions for each coordinate, for a total of 16^2^ unique TPMs. For each, it is possible to calculate the Φ spectrum that results using the algorithm we describe in the main text. Then, the upper bound on Φ is simply the maximum $\Phi^{MIP}$ value over all possible TPMs in all possible initial states. Performing this exercise results in the bound $\Phi^{MIP} \leq 1.5$, which is exactly one-fourth the analytical bound derived in the previous section; as discussed, it is likely that a factor of $1/2$ is accounted for if $D^{Max}(n)=n/2$, while the other factor of $1/2$ may be accounted for by the same type of argument applied to *ϕ*^*Max*^ (rather than *D*^*Max*^). If so, the upper bound on $\Phi^{MIP}$ would be $2^{n-4}n(n+1)$ and is potentially more tractable to calculate than previously believed.

### The PyPhi-Spectrum package

### Additional details related to the calculation of Φ values

In this section, we provide the transition probability matrices and initial states necessary to replicate our results (Tables A1–A11). The same data can be found in downloadable form via the GitHub repository: https://github.com/elife-asu/pyphi-spectrum.

**Table A1.** The TPM for a simple diode comprising two interconnected COPY gates taking input from one another such as that described in Chalmers and McQueen Chalmers and McQueen (2014).

s(t)	s(t+1)
00	00
10	01
01	10
11	11

**Table A2.** The TPM for an AND+OR system such as that described in Section “Methodology”.

s(t)	s(t+1)
00	00
10	01
01	01
11	11

**Table A3.** Simple electronic counter TPM from Hanson and Walker (2021).

s(t)	s(t+1)
000	110
100	000
010	101
110	010
001	100
101	111
011	001
111	011

**Table A4.** MAJ+MAJ+MAJ system from Figure 5.

s(t)	s(t+1)
000	000
100	000
010	000
110	111
001	000
101	111
011	111
111	111

**Table A5.** Fauré–Kaji binarization of the TPM for the p53-Mdm2 biological regulatory network from Gomez et al. (2021).

s(t)	s(t+1)
0000	1101
1000	1100
0100	1100
1100	1110
0010	1101
1010	1101
0110	1101
1110	1111
0001	0001
1001	0000
0101	0000
1101	0010
0011	0001
1011	0001
0111	0001
1111	0011

**Table A6.** The TPM for the entire Boolean network model of virus–host dynamics from Farnsworth (2021).

s(t)	s(t+1)
00000	00000
10000	00000
01000	10000
11000	10000
00100	01000
10100	01000
01100	11000
11100	11000
00010	00100
10010	00101
01010	10100
11010	10101
00110	01100
10110	01101
01110	11100
11110	11101
00001	00000
10001	00000
01001	10010
11001	10010
00101	01000
10101	01000
01101	11010
11101	11010
00011	00100
10011	00101
01011	10110
11011	10111
00111	01100
10111	01101
01111	11110
11111	11111

**Table A7.** The TPM for the reduced system from Farnsworth (2021).

s(t)	s(t+1)
000	000
100	000
010	100
110	101
001	010
101	010
011	110
111	111

**Table A8.** The TPM for the OR+AND+XOR system from Oizumi et al. (2014).

s(t)	s(t+1)
000	000
100	001
010	101
110	100
001	100
101	111
011	101
111	110

**Table A9.** The TPM for the MAJ+OR+AND+AND system from Tononi et al. (2016).

s(t)	s(t+1)
0000	0000
1000	0100
0100	0000
1100	1110
0010	0000
1010	1100
0110	1000
1110	1110
0001	0100
1001	0100
0101	0100
1101	1110
0011	0101
1011	1101
0111	1101
1111	1111

#### Photodiode Chalmers and McQueen (2014); Oizumi et al. (2014)

A photodiode is a simple system of two interacting COPY gates, taking input from one another. It is arguably the simplest “integrated” system one can study and has been studied in the context of IIT at least twice Chalmers and McQueen (2014); Oizumi et al. (2014). Following Chalmers and McQueen Chalmers and McQueen (2014), we set the initial state of the system to be $s_0=10$. The TPM is given below.

#### AND+OR  Hanson and Walker (2019); Albantakis et al. (2019)

Like the photodiode, the AND+OR system has been studied in the context of IIT at least twice prior to the current work Hanson and Walker (2019); Albantakis et al. (2019). However, a concrete Φ value is yet to be published. Therefore, we take the “published value” to be that of the PyPhi value found in Section “Methodology”. Similarly, we take the initial state to be $s_0=00$ in accordance with Section “Methodology”. The transition probability matrix is given below.

#### Hanson and Walker (2021)

This system is a three-bit digital counter in the initial state 101. The initial state is selected somewhat arbitrarily, since any initial state will work, but $s_0=101$ results in a particularly fast evaluation. The TPM, from Figure 4 of the original publication, is as follows:

#### Majority gate system

This system comprises three interconnected majority gates, each with three inputs, as shown in Figure 5. If the majority of inputs to a given node are 0, the state of the node at the next time step is $0,$ and if the majority of inputs to a given node are 1, the state of the node at the next time step is 1. In the main text, the system is evaluated in initial state $s_0=000$. The transition probability matrix is provided below.

**Table A10.** The TPM for the noisy AND+AND+AND+AND system from Hoel et al. (2016).

	0	1	2	3	4	5	6	7	8	9	10	11	12	13	14	15
0	0.24	0.10	0.10	0.04	0.10	0.04	0.04	0.02	0.10	0.04	0.04	0.02	0.04	0.02	0.02	0.01
1	0.24	0.10	0.10	0.04	0.10	0.04	0.04	0.02	0.10	0.04	0.04	0.02	0.04	0.02	0.02	0.01
2	0.24	0.10	0.10	0.04	0.10	0.04	0.04	0.02	0.10	0.04	0.04	0.02	0.04	0.02	0.02	0.01
3	0.00	0.00	0.00	0.00	0.00	0.00	0.00	0.00	0.00	0.00	0.00	0.00	0.49	0.21	0.21	0.09
4	0.24	0.10	0.10	0.04	0.10	0.04	0.04	0.02	0.10	0.04	0.04	0.02	0.04	0.02	0.02	0.01
5	0.24	0.10	0.10	0.04	0.10	0.04	0.04	0.02	0.10	0.04	0.04	0.02	0.04	0.02	0.02	0.01
6	0.24	0.10	0.10	0.04	0.10	0.04	0.04	0.02	0.10	0.04	0.04	0.02	0.04	0.02	0.02	0.01
7	0.00	0.00	0.00	0.00	0.00	0.00	0.00	0.00	0.00	0.00	0.00	0.00	0.49	0.21	0.21	0.09
8	0.24	0.10	0.10	0.04	0.10	0.04	0.04	0.02	0.10	0.04	0.04	0.02	0.04	0.02	0.02	0.01
9	0.24	0.10	0.10	0.04	0.10	0.04	0.04	0.02	0.10	0.04	0.04	0.02	0.04	0.02	0.02	0.01
10	0.24	0.10	0.10	0.04	0.10	0.04	0.04	0.02	0.10	0.04	0.04	0.02	0.04	0.02	0.02	0.01
11	0.00	0.00	0.00	0.00	0.00	0.00	0.00	0.00	0.00	0.00	0.00	0.00	0.49	0.21	0.21	0.09
12	0.00	0.00	0.00	0.49	0.00	0.00	0.00	0.21	0.00	0.00	0.00	0.21	0.00	0.00	0.00	0.09
13	0.00	0.00	0.00	0.49	0.00	0.00	0.00	0.21	0.00	0.00	0.00	0.21	0.00	0.00	0.00	0.09
14	0.00	0.00	0.00	0.49	0.00	0.00	0.00	0.21	0.00	0.00	0.00	0.21	0.00	0.00	0.00	0.09
15	0.00	0.00	0.00	0.00	0.00	0.00	0.00	0.00	0.00	0.00	0.00	0.00	0.00	0.00	0.00	1.00

**Table A11.** Three-node fission yeast TPM from Marshall et al. (2017).

s(t)	s(t+1)	s(t)	s(t+1)	s(t)	s(t+1)	s(t)	s(t+1)
0	2	128	162	256	78	384	110
1	2	129	162	257	66	385	98
2	130	130	162	258	2	386	162
3	130	131	162	259	2	387	162
4	4	132	132	260	76	388	76
5	0	133	128	261	68	389	68
6	128	134	128	262	4	390	132
7	128	135	128	263	0	391	128
8	8	136	136	264	76	392	76
9	0	137	128	265	72	393	72
10	128	138	128	266	8	394	136
11	128	139	128	267	0	395	128
12	12	140	140	268	76	396	76
13	0	141	128	269	76	397	76
14	128	142	128	270	12	398	140
15	128	143	128	271	0	399	128
16	256	144	384	272	332	400	332
17	256	145	384	273	320	401	320
18	384	146	384	274	256	402	384
19	384	147	384	275	256	403	384
20	260	148	388	276	332	404	332
21	256	149	384	277	324	405	324
22	384	150	384	278	260	406	388
23	384	151	384	279	256	407	384
24	264	152	392	280	332	408	332
25	256	153	384	281	328	409	328
26	384	154	384	282	264	410	392
27	384	155	384	283	256	411	384
28	268	156	396	284	332	412	332
29	256	157	384	285	332	413	332
30	384	158	384	286	268	414	396
31	384	159	384	287	256	415	384
32	18	160	178	288	82	416	114
33	18	161	178	289	82	417	114
34	146	162	178	290	18	418	178
35	146	163	178	291	18	419	178
36	16	164	144	292	84	420	84
37	16	165	144	293	80	421	80
38	144	166	144	294	16	422	144
39	144	167	144	295	16	423	144
40	16	168	144	296	88	424	88
41	16	169	144	297	80	425	80
42	144	170	144	298	16	426	144
43	144	171	144	299	16	427	144
44	16	172	144	300	92	428	92
45	16	173	144	301	80	429	80
46	144	174	144	302	16	430	144
47	144	175	144	303	16	431	144
48	272	176	400	304	336	432	336
49	272	177	400	305	336	433	336
50	400	178	400	306	272	434	400
51	400	179	400	307	272	435	400
52	272	180	400	308	340	436	340
53	272	181	400	309	336	437	336
54	400	182	400	310	272	438	400
55	400	183	400	311	272	439	400
56	272	184	400	312	344	440	344
57	272	185	400	313	336	441	336
58	400	186	400	314	272	442	400
59	400	187	400	315	272	443	400
60	272	188	400	316	348	444	348
61	272	189	400	317	336	445	336
62	400	190	400	318	272	446	400
63	400	191	400	319	272	447	400
64	66	192	194	320	78	448	78
65	66	193	194	321	66	449	66
66	130	194	130	322	66	450	194
67	130	195	130	323	66	451	194
68	68	196	196	324	76	452	76
69	64	197	192	325	68	453	68
70	128	198	128	326	68	454	196
71	128	199	128	327	64	455	192
72	72	200	200	328	76	456	76
73	64	201	192	329	72	457	72
74	128	202	128	330	72	458	200
75	128	203	128	331	64	459	192
76	76	204	204	332	76	460	76
77	64	205	192	333	76	461	76
78	128	206	128	334	76	462	204
79	128	207	128	335	64	463	192
80	320	208	448	336	332	464	332
81	320	209	448	337	320	465	320
82	384	210	384	338	320	466	448
83	384	211	384	339	320	467	448
84	324	212	452	340	332	468	332
85	320	213	448	341	324	469	324
86	384	214	384	342	324	470	452
87	384	215	384	343	320	471	448
88	328	216	456	344	332	472	332
89	320	217	448	345	328	473	328
90	384	218	384	346	328	474	456
91	384	219	384	347	320	475	448
92	332	220	460	348	332	476	332
93	320	221	448	349	332	477	332
94	384	222	384	350	332	478	460
95	384	223	384	351	320	479	448
96	82	224	210	352	82	480	82
97	82	225	210	353	82	481	82
98	146	226	146	354	82	482	210
99	146	227	146	355	82	483	210
100	80	228	208	356	84	484	84
101	80	229	208	357	80	485	80
102	144	230	144	358	80	486	208
103	144	231	144	359	80	487	208
104	80	232	208	360	88	488	88
105	80	233	208	361	80	489	80
106	144	234	144	362	80	490	208
107	144	235	144	363	80	491	208
108	80	236	208	364	92	492	92
109	80	237	208	365	80	493	80
110	144	238	144	366	80	494	208
111	144	239	144	367	80	495	208
112	336	240	464	368	336	496	336
113	336	241	464	369	336	497	336
114	400	242	400	370	336	498	464
115	400	243	400	371	336	499	464
116	336	244	464	372	340	500	340
117	336	245	464	373	336	501	336
118	400	246	400	374	336	502	464
119	400	247	400	375	336	503	464
120	336	248	464	376	344	504	344
121	336	249	464	377	336	505	336
122	400	250	400	378	336	506	464
123	400	251	400	379	336	507	464
124	336	252	464	380	348	508	348
125	336	253	464	381	336	509	336
126	400	254	400	382	336	510	464
127	400	255	400	383	336	511	464

#### Gomez et al. (2021)

This papers studies the p53-Mdm2 biological regulatory network. Typically, this network is multivalued, but there are two possible binarizations that make standard Φ calculations possible. Of these, we chose the Fauré and Kaji binarization as it is much faster to analyze than the Tonello binarization. Following the authors, we choose an initial state $s_0=0001$ and use the following TPM. Note that the PyPhi value we compute for this TPM differs from that published by the authors due to their use of several non-standard configuration settings, such as Krohn and Ostwalds definition of Φ as a difference in integrated conceptual information rather than the IIT 3.0 definition.

#### Farnsworth (2021)

In this paper, a virocell (virus-infected cell) is introduced into a Boolean network model of host cell dynamics. There are two network models provided, the first consists of five nodes and is the “full system”, while the second consists of three nodes and is the “reduced system”. For both systems, we study the case where all the nodes are “ON” (i.e. $s_0 = 11111$ and $s_0 = 111$, respectively). Following the Supplementary Material provided by Farnsworth, the transition probabilities matrices are given later. Note that, in the full system, the second node is an AND gate (as shown in Figure 6 of their main paper Farnsworth (2021)) rather than a COPY gate as shown in Figure 8 of their Supplementary Material.

#### Oizumi et al. (2014)

This is the canonical OR+AND+XOR system that is often used in demonstrating how to calculate Φ Tononi (2015); Oizumi et al. (2014); Mayner et al. (2018). Following Oizumi et al., we take the system to be in the initial state $s_0=100$. The transition probability matrix is given below.

#### Tononi et al. (2016)

This paper demonstrates the calculation of Φ for a simple system of four interacting logic gates: MAJORITY+OR+AND+AND. Following the authors, we use the initial state $s_0=1110$. The TPM is given below.

#### Hoel et al. (2016)

This paper examines several small Boolean networks at both micro- and macro-scales. We choose to analyze the smallest of microsystems here, which is a system of four interconnected AND gates with noisy input. Following the authors, we analyze the system in initial state $s_0=0000$. Due to the noisy input, the TPM is not deterministic and therefore cannot be written as an *N* by 2 matrix. Instead, it must be written as an *N* by *N* matrix where entry (*i*, *j*) specifies the probability of state *i* transitioning to state *j* at time step *t* + 1 (a standard TPM). The TPM is given below.

#### Marshall et al. (2017)

This model system is the fission yeast cell cycle from Marshall et al. (2017). As mentioned in the main text, we study the three node subsystem rather than the full eight-node subsystem (plus one external node) studied in the original publication. To calculate the spectrum of Φ values for this subsystem (or just the PyPhi Φ value), the TPM for the entire system (all nine nodes) is required. Therefore, there are 512 states in the TPM. Following the authors, the initial state of the system is set to $s_0=000110011$. In little-end binary notation (most significant bit on the right), the TPM used is as follows:

## Footnotes

1 see *e.g.*, citation tracking statistics for IIT and other theories using the SCOPUS database Burnham (2006)

2 Less than a day on a Mac Pro; 3.5 GHz 6-Core Intel Xeon E5 Processor

3 Implementation of the “Smallest”, “Biggest”, and “Moon” solutions are available via keyword arguments in the PyPhi-Spectrum package (Algorithm 3), while the KO17 solution is available by changing the USE-SMALL-PHI-FOR-CES option in the standard PyPhi configuration file.
